# Impact of gamma irradiation on physico-chemical and electromagnetic interference shielding properties of Cu_2_O nanoparticles reinforced LDPE nanocomposite films

**DOI:** 10.1038/s41598-024-54426-w

**Published:** 2024-02-20

**Authors:** Mohamad Bekhit, E. S. Fathy, A. Sharaf, M. Shiple

**Affiliations:** 1 Radiation Chemistry Department, National Center for Radiation Research and Technology, Egyptian Atomic Energy Authority, Cairo, Egypt; 2 Polymer Chemistry Department, National Center for Radiation Research and Technology (NCRRT), Egyptian Atomic Energy Authority (EAEA), Cairo, Egypt; 3 Radiation Engineering Department, National Center for Radiation Research and Technology (NCRRT), Egyptian Atomic Energy Authority (EAEA), Cairo, Egypt; 4 Electronic Engineering Department, National Telecommunication Institute, Cairo, Egypt

**Keywords:** Gamma radiation, Cu_2_O nanoparticle, LDPE, Nanocomposite, Shielding effectiveness (SE), Polymer chemistry, Chemistry, Materials science, Nanoscience and technology

## Abstract

In the current work, cuprous oxide (Cu_2_O) nanoparticles coated with Tween 80 were successfully synthesized via the chemical reduction method. Nanocomposites composed of low-density polyethylene (LDPE) and different ratios of Cu_2_O nanoparticles were fabricated by the melt mixing process. 10% of ethyl vinyl acetate (EVA) as a compatibilizing agent was added to the molten LDPE matrix and the mixing process continued until homogenous nanocomposites were fabricated. To study the influence of ionizing radiation on the fabricated samples, the prepared species were exposed to 50 and 100 kGy of gamma rays. The synthesized Cu_2_O nanoparticles were investigated by transmission electron microscopy (TEM) and X-ray diffraction (XRD). XRD and TEM analysis illustrated the successful formation of spherical Cu_2_O nanoparticles with an average size of 16.8 nm. The as-prepared LDPE/Cu_2_O nanocomposites were characterized via different techniques such as mechanical, thermal, morphological, XRD, and FTIR. Electromagnetic interference shielding (EMI) of the different nanocomposite formulations was performed as a promising application for these materials in practical life. The electromagnetic shielding effectiveness (SE) of the produced samples was measured in the X-band of the radio frequency range from 8 to 12 GHz using the vector network analyzer (VNA) and a proper waveguide. All the samples were studied before and after gamma-ray irradiation under the same conditions of pressure and temperature. The shielding effectiveness increased significantly from 25 dB for unirradiated samples to 35 dB with samples irradiated with 100 kGy, which reflects 40% enhancement in the effectiveness of the shielding.

## Introduction

Since decades ago, the polymeric materials have concerned great interest in many applications owing to their excellent characteristics as flexibility, ease of processing and high mechanical strength. The development of the polymeric materials is of great importance and obtained by forming composites through the addition of inorganic fillers. Polymeric composite materials are widely used in diverse fields such as materials used in transportation, construction, electronics, and consumer products. Recently, polymer nanocomposites are a new class in which the additives have extremely small phase dimensions, usually on the order of a few nanometers**.** The production of polymer nanocomposites for diverse applications in place of conventional materials is increasing exponentially due to light weight, cost efficiency and their remarkable physicochemical characteristics such as mechanical strength, electrical conductivity, thermal stability and biological applications^1–3^. Polyolefin polymers such as polyethylene are plastics of high commercial and economic importance as a result of their widespread use in all walks of life such as construction, electronics, sports, packaging and industrial applications. Low density polyethylene (LDPE) and ethylene vinyl acetate (EVA) polymer blends have been utilized in a wide range of engineering field because of their good physicomechanical properties. The addition of low quantity of EVA enhances the mechanical properties of LDPE and acting as a compatibilizer for improving the inorganic nanofiller loading and dispersion^4–7^.

Cuprous oxide (Cu_2_O) nanoparticles are important direct bandgap p-type metal oxide semiconductor (~ 2 eV) because of their wide range of potential applications such as solar energy conversion^8^, optical and magnetic materials^9^, gas sensing^10^, catalysis^11^, electrode materials^12^, pollutant adsorption^13^ and antimicrobial applications^14^. Moreover, Cu_2_O nanoparticles has gained a renewed interest for various technological applications due to its non-toxicity, economical, abundances of source materials, good environmental acceptability and its optoelectronic properties^15^. In addition, copper oxides possess unique dielectric properties, which can become a promising electromagnetic (EM) shielding and wave-absorbing material^16–19^. Currently, there are many well- known preparation methods for Cu_2_O nanoparticles such as chemical reduction^20^, thermal oxidation of Cu metal^21^, laser ablation^22^, thermal decomposition^23^, microemulsion^24^, microwave irradiation^25^, electro-deposition^26^ and microplasma method^27^.

Treating polymers with ionizing radiation (gamma rays, accelerated electrons, ion beams, and X-rays) is a promising technology for producing advanced polymeric materials. This technology is considered safe because it does not require solvents or initiating materials at high temperatures. Ionizing radiation exposure for polymeric matrix has the ability to produce excited species and free radicals (primary and secondary) that can be transformed into into various paths as disproportion, hydrogen abstraction, arrangements and/or the creation of new other bonds. These events finally cause crosslinking and/or degradation of polymeric materials depending on the exposed irradiation doses. Briefly, ionizing radiation is a clean technique that is considered a basis of the reaction which leads to an initiation and cross-linking between chains with further a sterilization procedure for the polymeric materials^28–32^.

Recently, the protection from electromagnetic wave pollution has received great attention due to the problems of interference between electronic devices and its threat to human health. Electromagnetic shielding is described as reducing the propagation of electrical and magnetic waves from one area to another by using electrically conductive and/or magnetic materials^33^. Because of the accelerated growth of telecommunications, electrical, and electronic systems, the study of these kinds of materials has consider of great significance to limit the spread of electromagnetic interference (EMI). Some examples of the effects of electromagnetic interference are harsh interruption of electronic or remotely controlled devices, generation of false images (radar), and deterioration of the efficiency, lifespan and safety of electrical equipment. Electromagnetic shielding can be accomplished by reducing the propagated signals that crosses a region, either by reflection of the wave or by absorption and dissipation. Two main material categories are used to achieve the required electromagnetic shielding; metal and non-metal elements. Metal based material; steel, copper, nickel and aluminum with different types such as sheets, screens or foams show negative properties; high density, poor resistance to corrosion, cost processing. Non-metal based system; polymer composites containing conductive nanomaterials show the best alternative since it solves all problems of metal based material besides their positive characteristic such as excellent mechanical features, thermal stability, lightness and corrosion resistance.

The work aims to synthesize Cu_2_O as nanofillers in the LDPE matrix. The LDPE/Cu_2_O nanocomposites were investigated and examined as electromagnetic interference shielding material.

## Experimental

### Materials

Copper sulfate pentahydrate was obtained from El-Goumhouria Co., Cairo, Egypt. Ascorbic acid was obtained from Merck Chemical Co., Germany. Tween 80 surfactant (T80) was obtained from MP Biomedical Co., India. Low density polyethylene pellets were obtained from El Sewedy Plastic Manufacturing (SEDPLAST), Tenth of Ramadan City, Cairo, Egypt. Ethylene vinyl acetate containing 18% of vinyl acetate was obtained from Arkema Inc., North America. Bidistalled water was utilized throughout the preparation steps.

### Preparation of Cu_2_O nanoparticles

Cu_2_O nanoparticles were prepared by using aqueous solution reduction method with ascorbic acid as a reducing agent^34^**.** Firstly, CuSO_4_ (0.015M) was dissolved in a T80 solution (0.5 wt% in water) under a magnetic stirrer at 65 °C for 30 min. After that, ascorbic acid (0.15M) was added into the CuSO_4_/T80 solution at 65 °C under continuous stirring, and then the solution pH value was raised and adjusted to pH 12 by using 2 M NaOH solution. After 30 min, the solution color changed to an orange colloid confirming the successful preparation of Cu_2_O nanoparticles. Cu_2_O nanoparticles were separated by centrifugation at 6,000 rpm and washed several times with water–ethanol solution, and then dried at room temperature for 24 h.

### Fabrication of LDPE/Cu_2_O nanocomposites films

**Figure 1:**
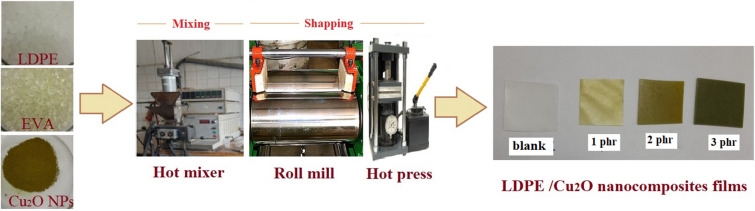
Preparation steps of LDPE/Cu_2_O nanocomposites films.

Nanocomposites films of LDPE containing Cu_2_O nanoparticles were prepared by melt blending process using a laboratory mixer (Plasticorder model PL-2100; Brabender, Germany)). Melt blending technique is a cost-effective technique and widely used in the industry. Firstly, for melting of LDPE pellets, it injected in the hot mixer at temperature nearly 165 °C for 5 min^35^**.** After that, 10% of EVA as a compatibilizing agent was added into the molten LDPE with continued mixing for a further 5 min at the same temperature to achieve complete homogeneous mixing. Then, the nanocomposites were formed by mixing different concentrations (0, 1, 2, and 3 part per hundered resin (phr)) of Cu_2_O nanoparticles into the LDPE/EVA matrix at a rotor speed of 60 rpm for 5 min. Then, the nanocomposites were quickly taken from the mixer to an open roll mill to obtain straight films that are easy to press. Polymeric sheets of 1.0 mm thickness were formed by hot pressing at 165 °C / 5 min (2 min preheating and 3 min at 15 MPa pressure). The molded plastic sheets were cooled by water-cooled presser at the same pressure (Fig. 1). Finally, to study the impact of ionizing radiation, the formed nanocomposites films were gamma irradiated to 50 and 100 kGy. The irradiation process occur using ^60^Co facility at a constant dose rate (0.8 kGy/h) at room temperature in the gamma radiation unit that present in National Centre for Radiation Research and Technology (NCRRT); Egyptian Atomic Energy Authority (EAEA), Egypt.

### Measurements

The X-ray diffraction (XRD) analysis the synthesized Cu_2_O nanoparticles and LDPE/Cu_2_O nanocomposites films was performed using an X-ray diffractometer (Shimadzu 6000, Tokyo, Japan) equipped with a Cu Kα (1.5418 Å) X-ray source. Both size and shape of the synthesized Cu_2_O nanoparticles was observed by Transmission electron microscopy (TEM) (a JEOL JSM-100 CX model instrument worked at 80 kV accelerating voltage). The infrared (IR) spectra LDPE/Cu_2_O nanocomposites films were measured using Attenuated total reflectance-Fourier transform infrared (ATR-FTIR) apparatus (Bruker Vertex70, Germany) within the spectral range from 500 to 4000 cm^−1^. The surface morphology of LDPE/Cu_2_O nanocomposites films was observed by scanning electron microscope (SEM) (ZEISS EVO-15, UK) operated at an acceleration voltage of 30 kV. For the SEM measurement, the fractured surfaces were coated with a thin layer of gold in order to avoid electrical charging under the electron beam. For obtaining the mechanical analysis of LDPE/Cu_2_O nanocomposites films, a dumbbell-shaped examination sections were measured at 300 mm/min of crosshead speed via a tensile testing machine (Qchida computerized testing instrument; Dongguan Haida Equipment Co. Ltd; China). The ISO 527-2 was detected. The average value of the mechanical factors was taken via at least three testers. Thermogravimetric (TG) analysis was performed using a Shimadzu TGA-50 (Kyoto, Japan) to study the thermal stability of nanocomposites. The temperature monitored from ambient to 600 °C at a heating rate of 10 °C/min with a nitrogen flow of 20 mL/min. Direct current (DC) conductivity measurements for LDPE/Cu_2_O nanocomposites films were carried out at room temperature. The sample was positioned in a conductivity measuring cell in a sandwich configuration. HP 4280A C-V Plotter (USA) was used for measuring the conductivity of the samples under test.

### Electromagnetic interference assays

**Figure 2:**
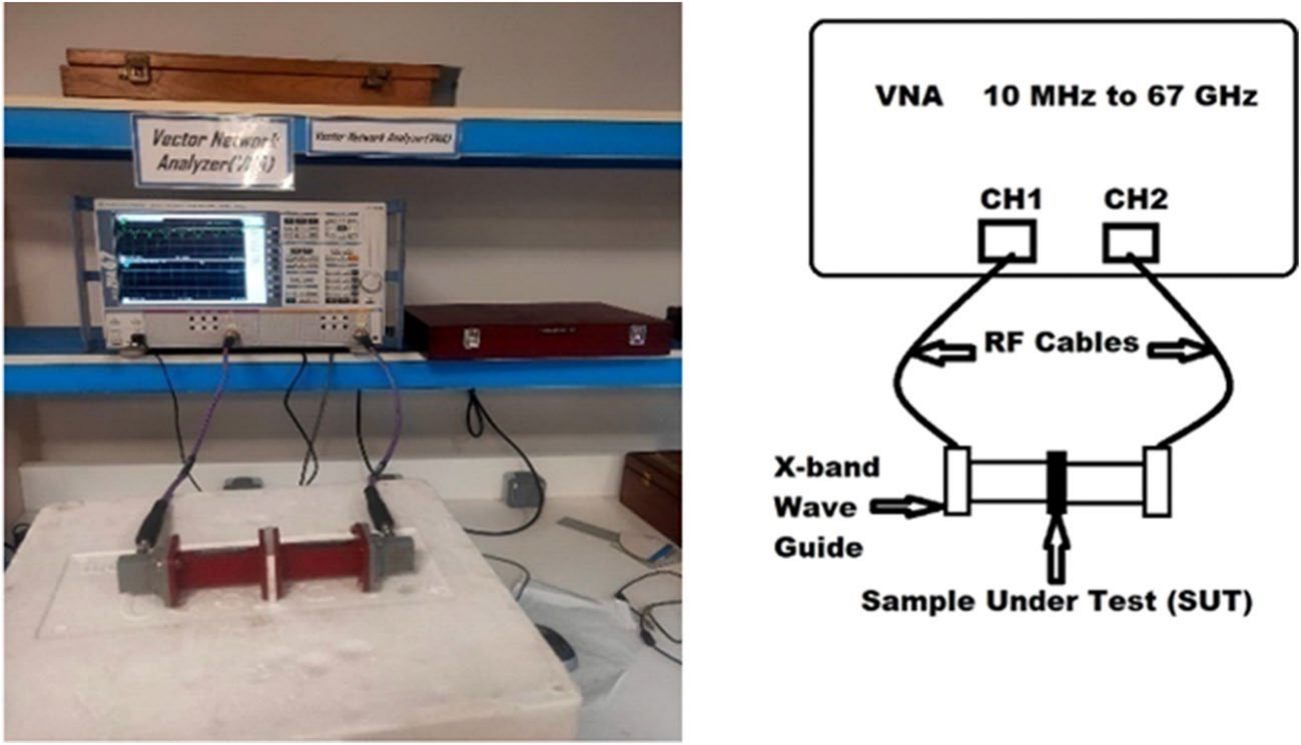
The measuring setup using the vector network analyzer.

Shielding effectiveness for the unirradiated and irradiated nanocomposites with gamma doses of 50 and 100 KGy was measured using a vector network analyzer and a proper wave guide. This measurement uses the R&S ZVA 67 VECTOR NETWORK ANALYZER operates in the range 10 MHz to 67 GHz and a wave guide operates in the X-band from 8 to 12 GHz. The VNA manufactured in Germany by Rohde & Schwarz GmbH & Co KG. The measuring setup was shown schematically in Fig. 2.

## Result and discussion

### Characterization of Cu_2_O nanoparticles

#### X-ray diffraction analysis of Cu_2_O nanoparticles

**Figure 3:**
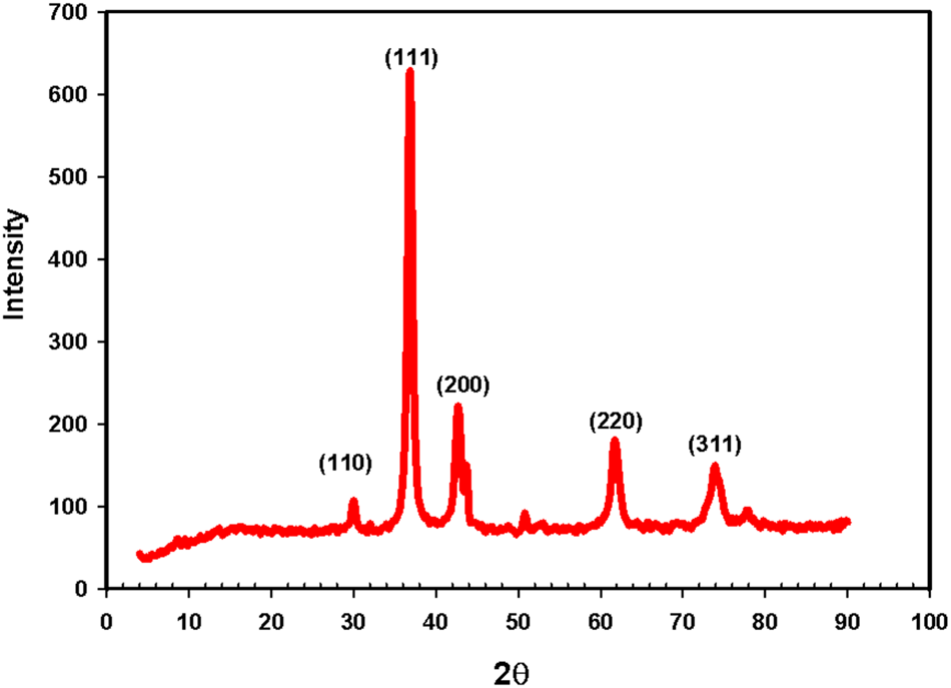
XRD patterns of Cu_2_O nanoparticles.

The XRD analysis is an indispensable step in gaining information about the crystal structure and phase analyses of nanomaterials. Figure 3 represents the XRD peaks of Cu_2_O nanoparticles. The XRD spectrum of the Cu_2_O nanoparticles showed the distinctive diffraction peaks observed in the spectra at 30.01, 36.88, 42.72◦, 61.88◦, and 73.96◦ correspond to the crystal planes (110), (111), (200), (220) and (311), respectively, of the cubic phase of cuprous oxide (Cu_2_O)^36^**.** Also, the sharp diffraction peaks of Cu_2_O nanoparticles indicating that these Cu_2_O nanoparticles have high crystallinity. The crystallite size of Cu_2_O nanoparticles (D) was considered based on the main plane of (111) using Scherrer formula (D = kλ/βcosθ), where k, λ, β and θ are the shape or geometry factor (k = 0.9), X-ray wavelength (λ = 0.1541 nm), the full width at half maximum (FWHM) of diffraction peak and the diffraction angle, respectively. Using the FWHM of the strong and sharp diffraction peak (111), the crystallite size was found to be approximately 13.08 nm.

#### TEM analysis of Cu_2_O nanoparticles

**Figure 4:**
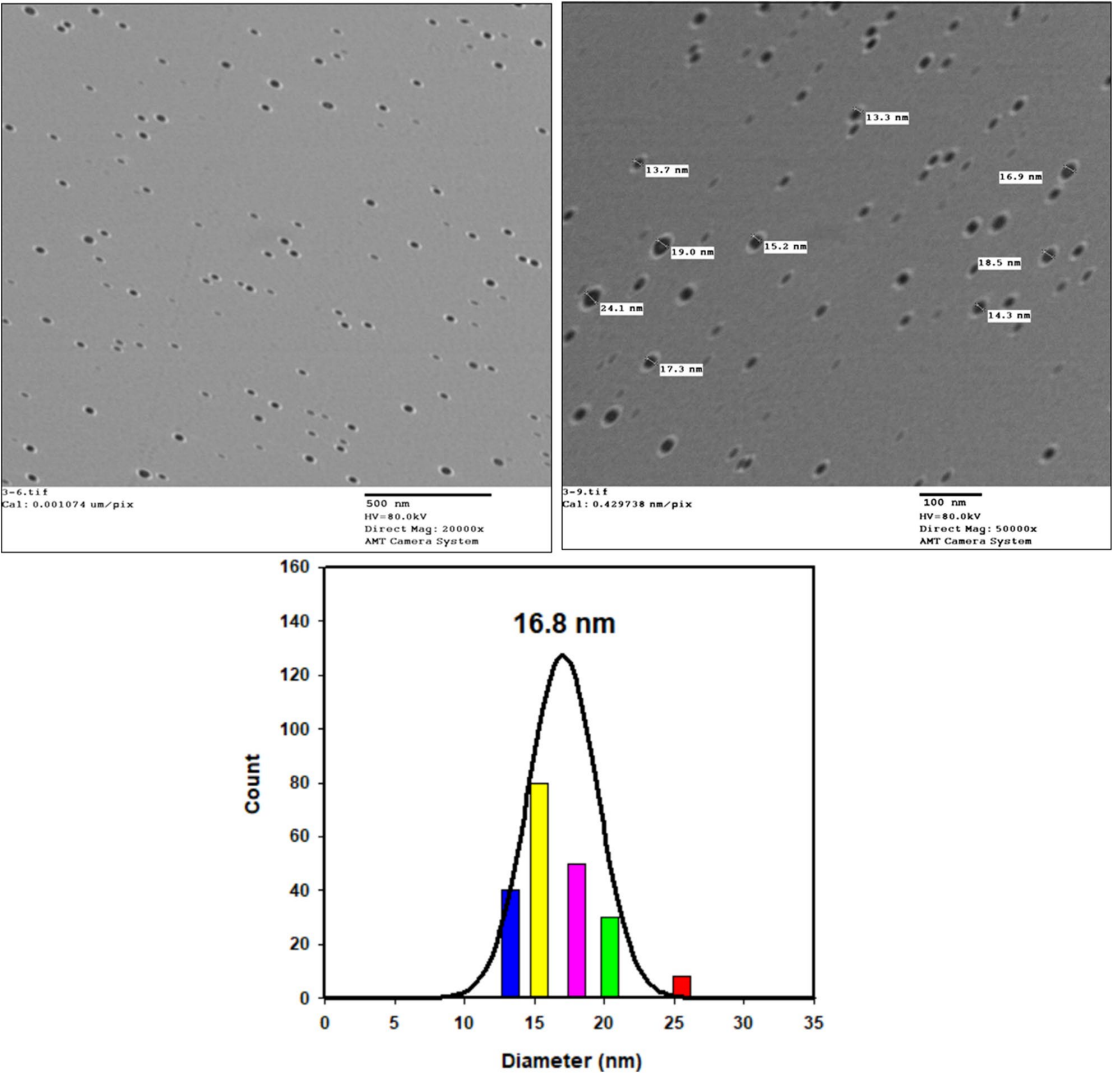
TEM image with different magnifications and the particle size distribution by Gaussian fitting of Cu_2_O nanoparticles.

Both shape morphology and particle size of the Cu_2_O nanoparticles were explained by TEM and shown in Fig. 4. It can be observed clearly that the Cu_2_O nanoparticles have uniform spherical-shaped particles. Also, it is observed that the prepared Cu_2_O nanoparticles are set individually dispersed, as in TEM photo, which signifying the protective role of T80. Also, a narrow size distribution histogram of Cu_2_O nanoparticles revealed an average diameter approximately at 16.8 nm and this result is matched with XRD result.

### Characterization of LDPE/Cu_2_O nanocomposite films

#### Mechanical measurements

**Figure 5:**
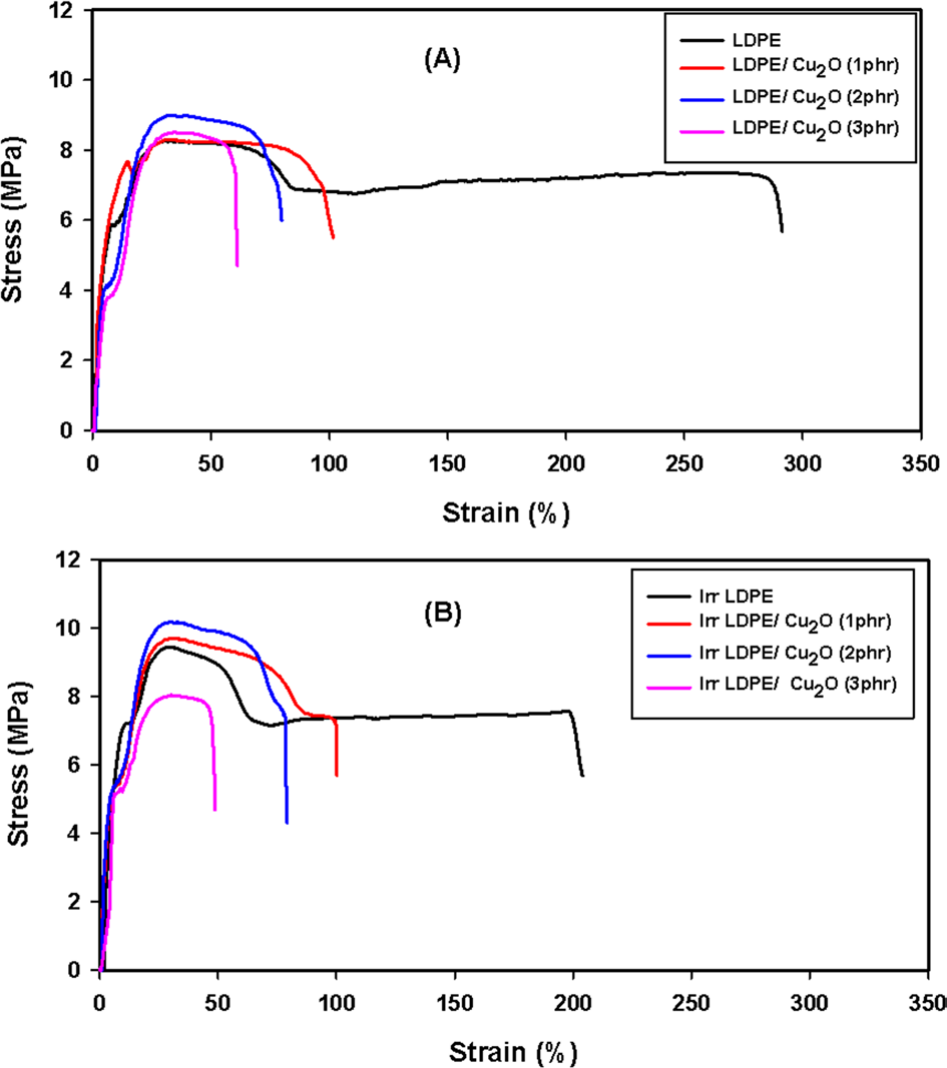
Stress–Strain curve of (**A**) unirradiated LDPE/Cu_2_O and (**B**) Irradiated LDPE/Cu_2_O nanocomposites at 100 kGy.

The stress–strain curve is displayed in Fig. 5A,B. As clear, the stress of LDPE increased with Cu_2_O nanoparticles, at the same time the nanocomposite reinforced with 2 phr of Cu_2_O clear superiority about the other for each un-irradiated and irradiated sample. Whereas, the strain of the nanocomposites was reduced with nanoparticle interface due to the rigidity and stiffness brought into LDPE texture. Furthermore, the irradiation dose declined the strain due to the restricted mobility caused by radiation-induced crosslinking effect.

**Figure 6:**
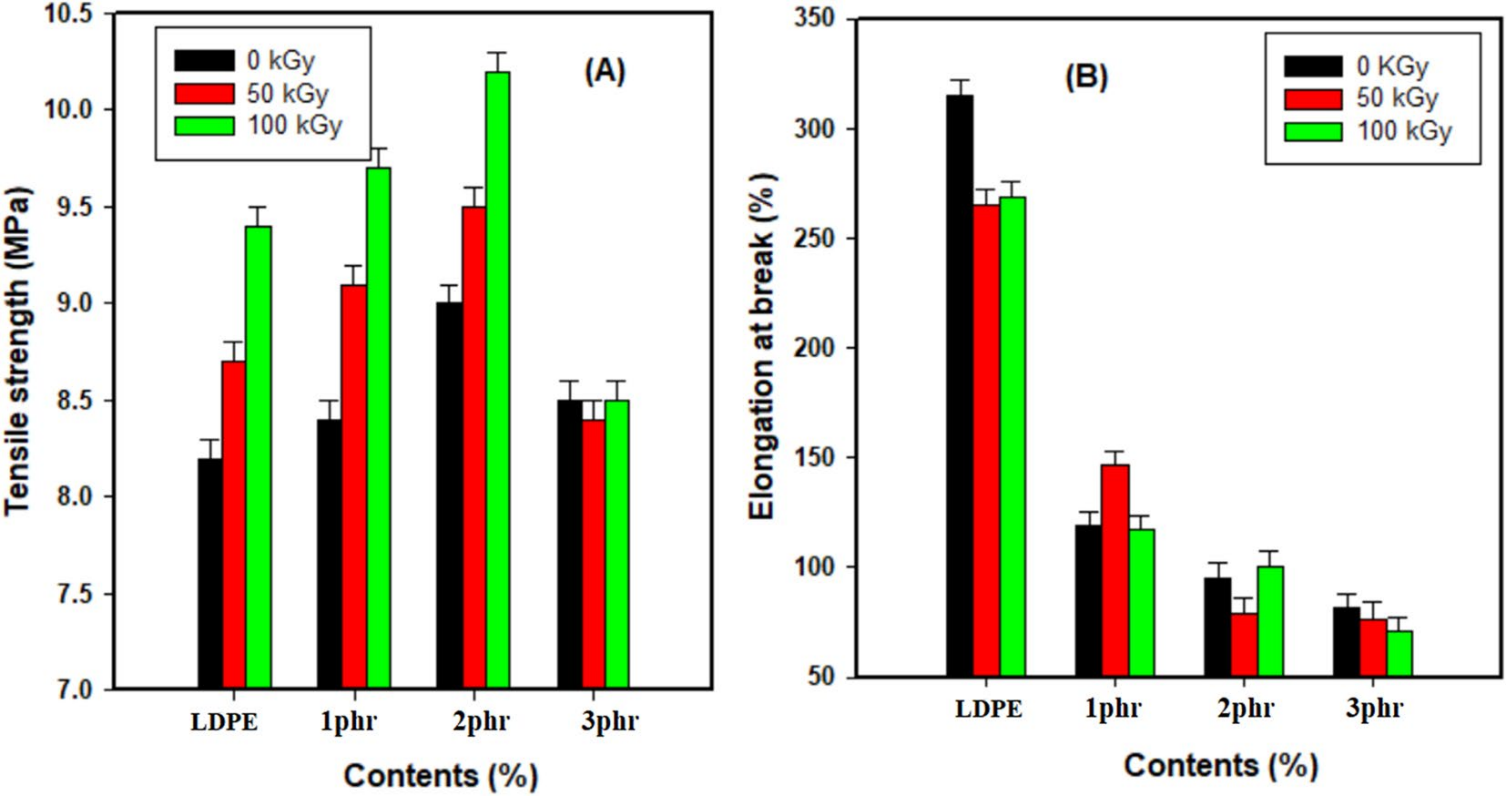
**(A)** Tensile strength (MPa), (**B**) Elongation at break (%) of LDPE and LDPE/Cu_2_O nanocomposites exposed to different irradiation doses.

The explanation of the previous stress–strain curve through the studying of the tensile strength (TS), and elongation at break (E%) were implemented for the fabricated sheet samples of the LDPE and LDPE/Cu_2_O nanocomposite specimens, respectively, as specified in Fig. 6. A low content of a dispersed additive (up to 2.0 phr) could develop the tensile properties of LDPE (Fig. 6A). This phenomenon is credited to the uniform distribution of additive nanoparticles^37,38^**.** Controlling the concentration of distributed fillers is established on the reduction in strength property of materials at concentrations above the stated upper threshold values. If the concentration of additive or filler surpasses the threshold values, an accumulation of particles happens in the polymeric matrix, leading to a decline in strength features. This observation was achieved when the percentage of the Cu_2_O nanoparticles was 3.0 phr^38^**. **On the other hand, as the radiation dose increases from 50 to 100 kGy more crosslinking is created in the polymeric chains leading to increase in TS values and also the synergistic effect of both irradiation doses and filler contents up to 2.0 phr lead to the enhancement of the tensile strength values. Consequently, the two applied irradiation doses and interface of nanoparticle up to 2.0 phr improved the TS of polymer matrix due to the synergism effect between them.

From Fig. 6B, Inverse effects were predominant in case elongation at break studies caused by nanofiller and radiation doses. The reduction in elongation at break with rising filler contents can be ascribed to the restriction in mobility of polymer chains that occurred by adhesion and interaction of nanofiller that did not allow the polymer chains to move causing a decrease in elongation^39^**.** On the other hand, the decrease in elongation at break with rising radiation doses is credited to the radiation-induced crosslinking effect^40^. The crosslinking cause the binding of adjacent polymeric chains and consequently the molecular mobility is hindered and a rupture for polymeric chains takes place at lower elongation value^41^.

#### FTIR investigation

**Figure 7:**
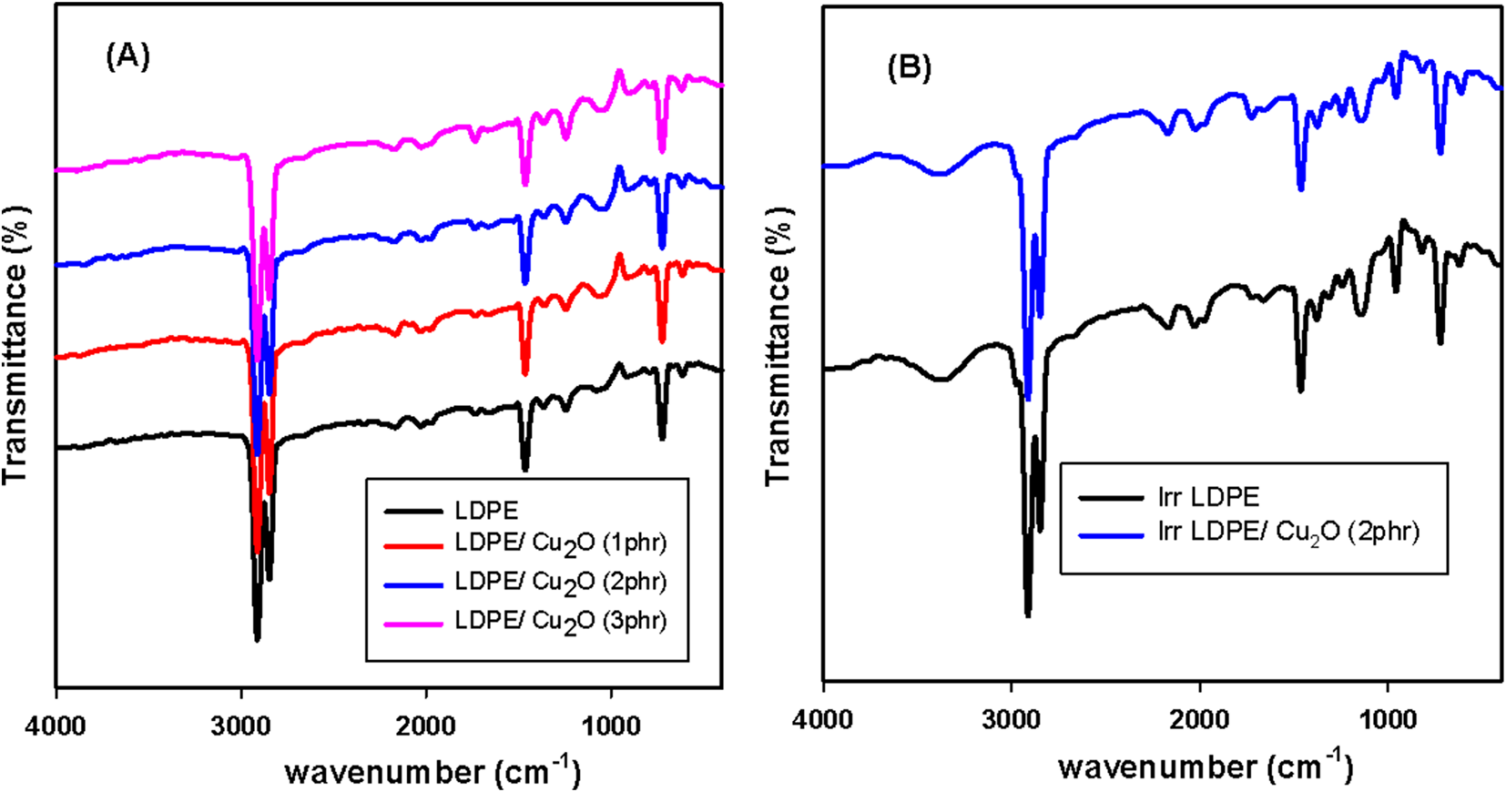
FTIR of (**A**) LDPE reinforced with differnt concentrations of Cu_2_O nanoparticles. (**B**) LDPE and LDPE/(2 phr) Cu_2_O nanocomposite irradiated 100 kGy.

In the spectral range of 4000–500 cm^−1^, bands of the FTIR were stately by plotting a graph of wave number (cm^-1^) against transmittance (%). Figure 7A was listed to recognize the probable interface between the LDPE/EVA matrix and Cu_2_O nanoparticles at various percentage loading. From Fig. 7A several bands are distinct to the successful blending of LDPE and EVA such as, CH_2_ stretching at 2920 cm^−1^ and its bending vibration at 615 cm^−1^ which corresponds to LDPE and EVA. Furthermore, the band at 1745 cm^−1^, matches the C = O stretching of the EVA acetate group. After interfacing of Cu_2_O nanoparticles into the LDPE matrix, the FTIR of the strengthened nanocomposites doesn’t show evident alterations in the FTIR spectra of the LDPE matrix reflecting the physical interaction of Cu_2_O nanoparticles inside LDPE matrix^42^. Figure 7B represents the FTIR of irradiated LDPE and its nanocomposite loaded with 2 phr Cu_2_O nanoparticles as this percent recorded the best mechanical properties. The peak intensity of carbonyl group at 1745 cm^-1^ slightly increased after irradiation due to the occurrence of oxidative phenomena during irradiation and formation of the carbonyl group^43^. After irradiation, OH broad band appear at 3400 cm^-1^ in LDPE and its nanocomposite are due to the presence of oxygen surrounding in gamma irradiation cell and occurrence of some chain scission^44^.

#### XRD of LDPE/Cu_2_O nanocomposite

**Figure 8:**
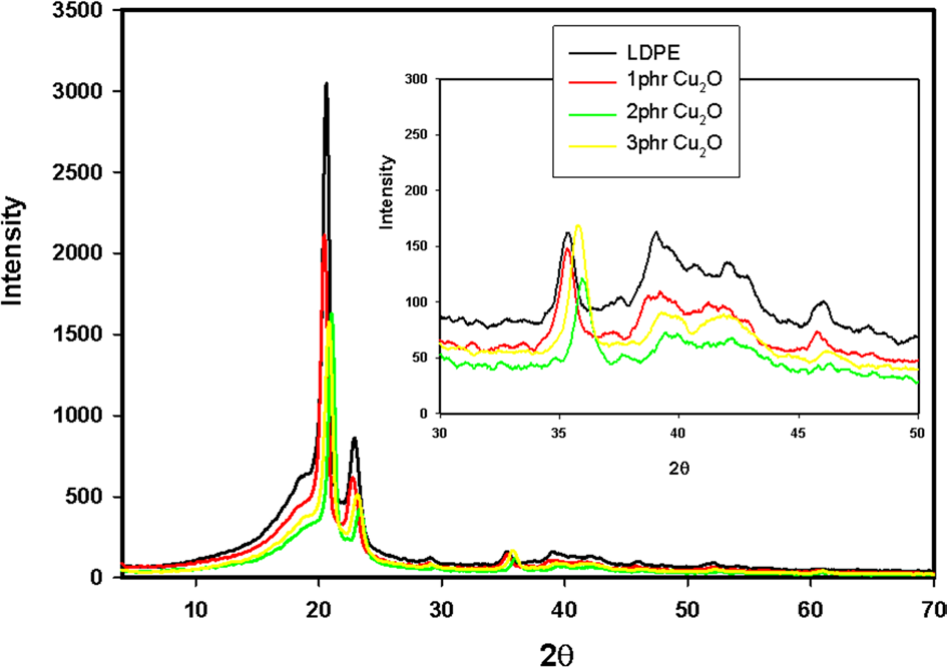
XRD patterns of LDPE and LDPE/Cu_2_O nanocomposite with different concentrations.

**Figure 9:**
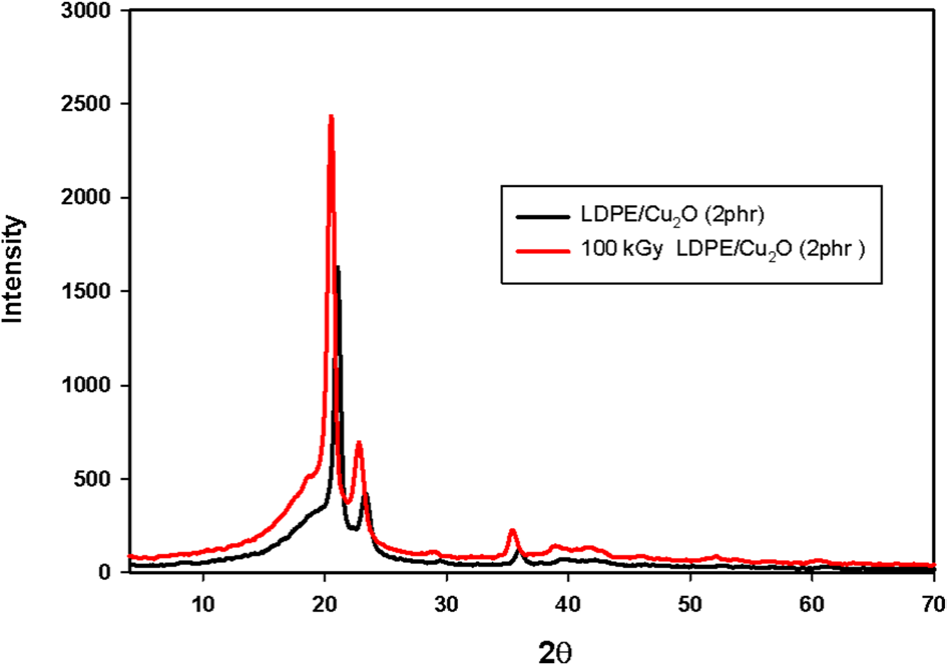
XRD patterns of unirradiated and 100 kGy irradiated LDPE/Cu_2_O (2 phr) nanocomposite.

Figure 8 depicts the XRD patterns of pristine LDPE and LDPE/Cu_2_O nanocomposite with different concentrations of Cu_2_O nanoparticles. The peaks at 2θ =20.6°, 22.8°, 29.1°and 35.4° are assigned to the (110), (200), (210) and (220) lattice planes of LDPE, respectively^45^. Upon addition of Cu_2_O nanoparticles into LDPE matrix, the diffraction peak intensity of LDPE was reduced due to the decrease in the crystallinity. This result supported the good interfacial interaction between the Cu_2_O nanoparticles and the polymer chains with the formation of homogeneous nanocomposite^39^. No diffraction peaks corresponding to Cu_2_O nanoparticles are observed in the LDPE nanocomposites due to their low concentrations^42^. Moreover, the shifting occurs for the peak at 36° of LDPE is due the interference with main peak of Cu_2_O nanoparticles. On the other hand, irradiated LDPE/Cu_2_O nanocomposite at 100 kGy displayed in (Fig. 9) showed an enhancement in crystallinity. This consequence is qualified to crosslinking effect of gamma radiation^45,46^.

#### Thermogravimetric analysis (TGA)

**Figure 10:**
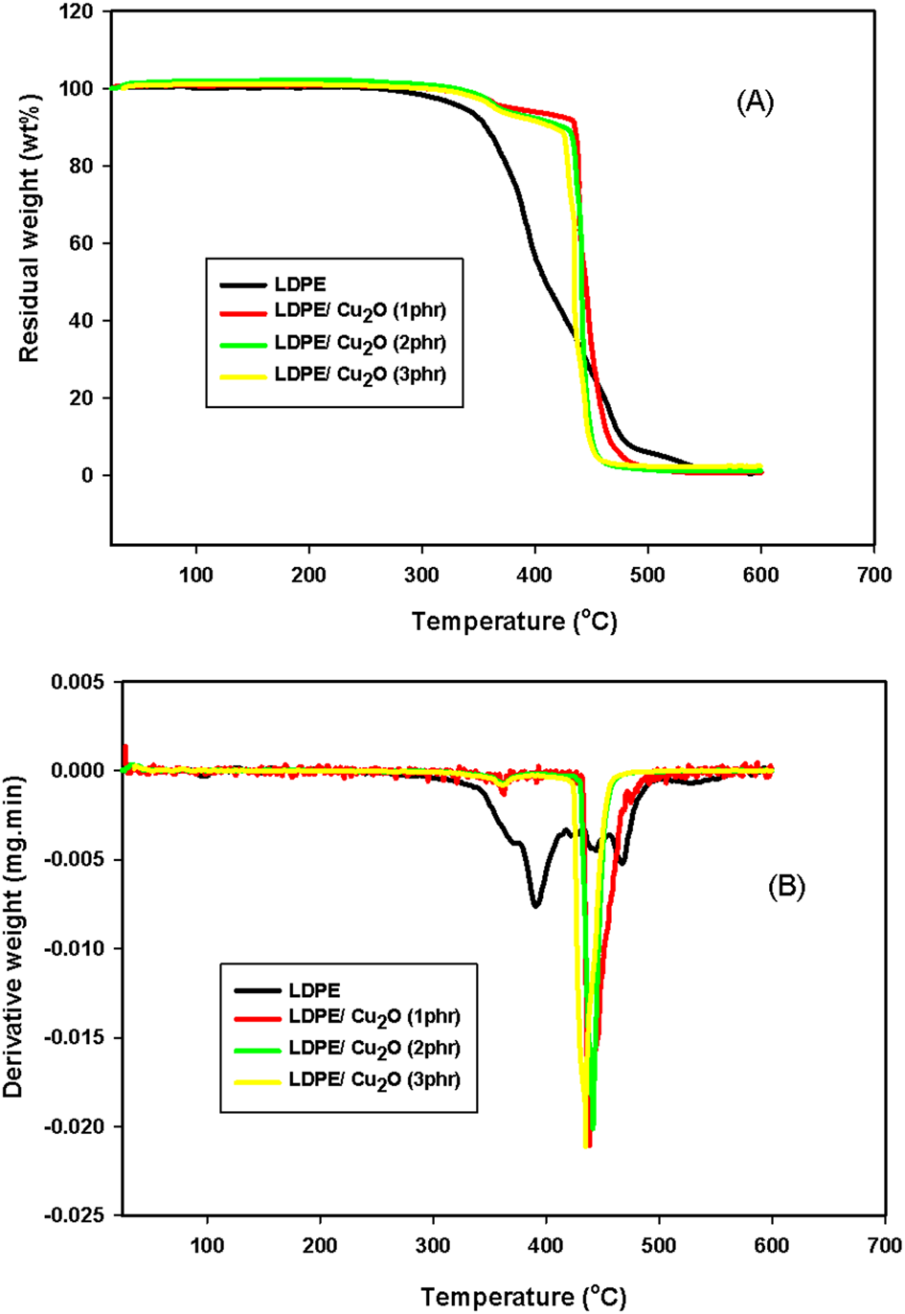
(**A**) TGA and (**B**) DTG of LDPE, LDPE/1 phr Cu_2_O, LDPE/2 phr Cu_2_O and LDPE/3 phr Cu_2_O nanocomposites.

The TGA investigation is a characteristic procedure in which alterations in the mass are detected as the sample is progressively heated. The thermal stability of LDPE reinforced with different ratios of Cu_2_O nanoparticle is measured and displayed in Fig. 10 and the several degradation stages are itemized in Table 1. By following the TGA curves exhibited in Fig. 10 and the mass loss values recorded in Table 1, the values show that the decomposition stages of the nanocomposite mass loss mainly depend on the Cu_2_O filling and applied radiation dose. It is apparent that the LDPE/Cu_2_O nanocomposite's thermal stability clearly improved with all Cu_2_O percentages. To examine the magnitude of LDPE thermal stability affected by Cu_2_O nanoparticles, wherein the different temperature mass loss, Tml_10_, Tm_25_, Tm_50_, and Tm_75_ and residual weight at 600 °C of the native LDPE recorded respectively, 365 °C, 382 °C, 409 °C, 452 °C, and 0.6%. These values shifted to higher temperature mass loss by incorporating 2 phr of Cu_2_O as an example, reflecting the thermal stability of the polymer matrix which was arranged respectively as follows, 426 °C, 437 °C, 441 °C, 444 °C, and residual weight at 1.2%.

**Table 1:** TGA parameters of LDPE-Cu_2_O nanocomposites irradiated at 100 kGy.

LDPE/Cu_2_O formulations (wt%)	Dose (kGy)	Tml_10_ (^o^C)	Tml_25_ (^o^C)	Tml_50_ (^o^C)	Tml_75_ (^o^C)	Residual weight at 600 (^o^C)
LDPE	0	356	382	409	452	0.6
	100	339	370	425	462	0.1
LDPE/1.0 phr Cu_2_O	0	435	438	444	453	0.8
LDPE/2.0 phr Cu_2_O	0	426	437	441	444	1.2
	100	411	419	432	452	1.4
LDPE/3.0 phr Cu_2_O	0	415	430	434	441	2.4

**Figure 11:**
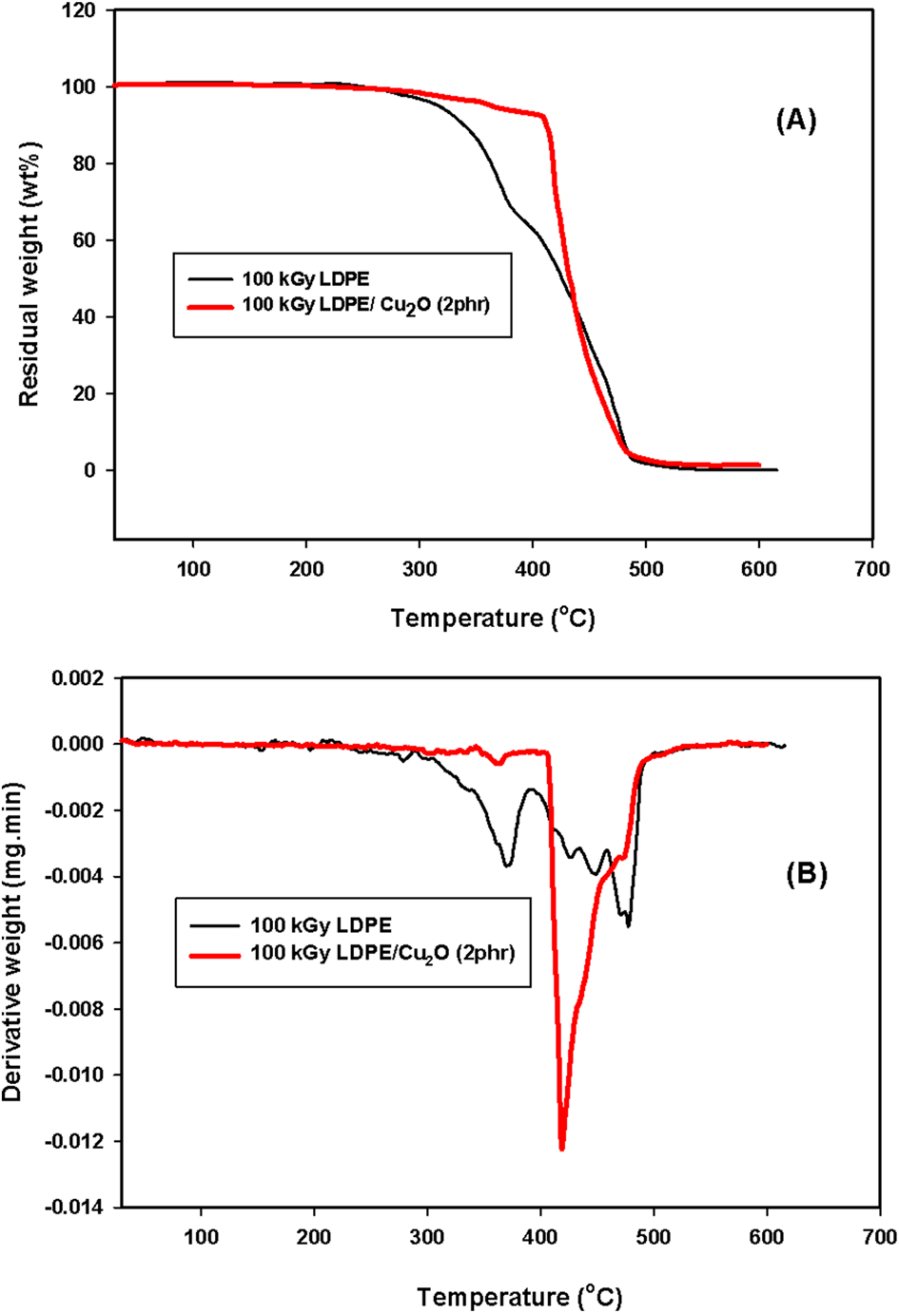
(**A**) TGA and (**B**) DTG of LDPE and LDPE/2 phr Cu_2_O nanocomposite irradiated at 100 kGy.

We selected LDPE/ Cu_2_O (2 phr) nanocomposite as the best component that had achieved good mechanical properties to study the effect of irradiation dose on its thermal stability (Fig. 11). Obviously, the thermal stability of native irradiated LDPE was decreased at the early stages of decomposition (Tm_10_ and Tml_25_) due to the release of volatile compounds and water vapor. On the other hand, at lately stages of the decompositions (Tm_50_ and Tm_75_), it shifted to a higher value when exposed to gamma irradiation. This is credited to the effect of gamma irradiation and crosslinking density creation inside the LDPE matrix. Furthermore, for irradiated LDPE and LDPE/ Cu_2_O (2 phr) nanocomposite, the superior thermal stability of nanocomposite reflect the synergistic impact of both nanoparticle and gamma irradiation on the thermal stability of the pristine LDPE.

#### Scanning electron microscope

**Figure 12:**
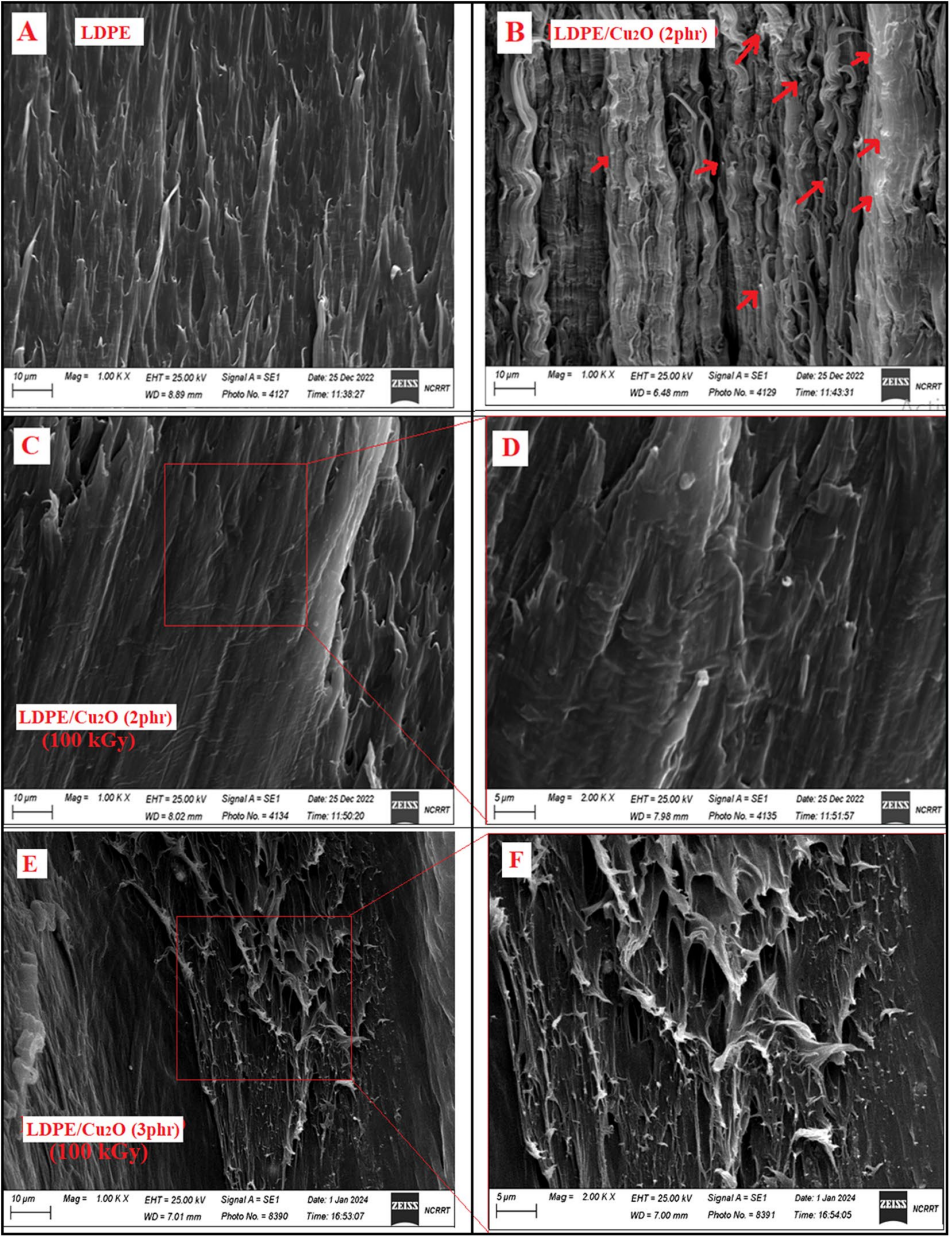
SEM images of (**A**) pristine LDPE, (**B**) Unirradiated LDPE/Cu_2_O (2 phr) nanocomposite, (**C**, **D**) Irradiated LDPE/Cu_2_O (2 phr) nanocomposite with different magnification and (E–F) Irradiated LDPE/Cu_2_O (3phr) nanocomposite with different magnification.

The morphology of the LDPE and the unirradiated and gamma irradiated nancomposites with Cu_2_O (2 phr) as nanofillers is shown in the SEM cross section representative images of Fig. 12. As shown in Fig. 12A, the unirradiated LDPE film has a roughness surface. Figure 12B show a rigid surface due to the homogeneous dispersion of the individual nanofillers and no large agglomerated are detected in the sample. As shown in Fig. 12C, the surface roughness of the gamma irradiated LDPE/Cu_2_O (2 phr) nanocomposite film decreases with the sample irradiation indicating radiation crosslinking process. It can be observed that, Cu_2_O nanoparticles are uniformly dispersed and sphere-like structures. With increasing the concentration of Cu_2_O nanoparticles (3phr), there is high degree of surface roughness and aggregation of Cu_2_O nanoparticles inside LDPE polymer matrix (Fig. 12D).

#### Conductivity of nanocomposites

**Figure 13:**
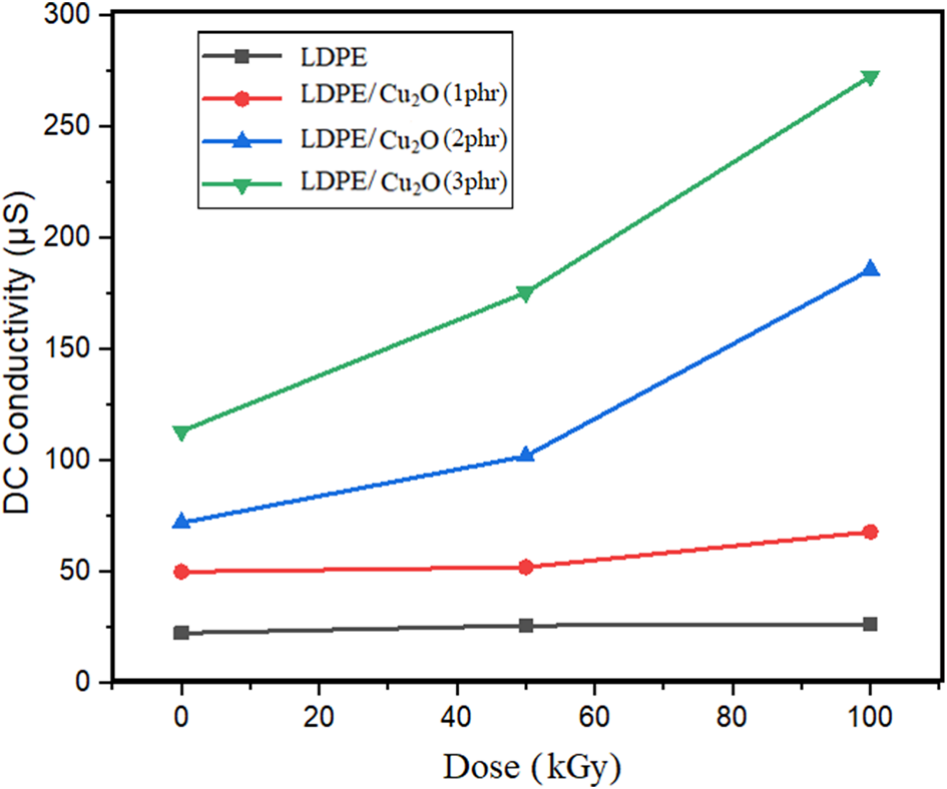
DC conductivity of LDPE films with different concentrations of Cu_2_O nanoparticles at various gamma irradiation doses.

Figure 13 indicates the conductivity characteristics of LDPE films with different concentrations of Cu_2_O nanoparticles and gamma irradiations. It can be seen that the conductivity of LDPE increases with the incorporation of Cu_2_O nanoparticles. Also, the conductivity enhanced significantly with increasing gamma-irradiation doses from 50 to 100 kGy. Abdel Moez et al. studied the impact of gamma radiation on LDPE films and found that the direct energy gap decreases with increasing radiation doses and their results ascribed to the radiation effect that increases the number of free electrons which enhance the electric conductivity significantly^47^. Also, Elnaggar et al. (2023)^38^, Abdel Maksoud et al. (2021)^48^, Tommalieh (2021)^49^ and found that gamma radiation decreases the energy band gap of polymer/metal oxide nanocomposites due to the increase the number of energy-localized electronic states between the valence and conduction bands related to the subjection to gamma radiation where the chains becoming more and more cross-linked with one another as a result of subsequent irradiation **.**

### Electromagnetic shielding effectiveness

The ability of a material to attenuate the propagation of an incident electromagnetic wave defines the concept of electromagnetic shielding perfectly. The attenuation of these waves may be due to reflection absorption and even if multiple reflections. The power ratio between the incident and transmitted electromagnetic waves represents its shielding effectiveness (SE). The total shielding effectiveness (SE_TOT_) is computed as the sum of the reflected shielding effectiveness (SE_R_), the absorption shielding effectiveness (SE_A_), and the multiple shielding effectiveness (SE_MR_),which can be written as: *SE*_*TOT*_ = *SE*_*R*_ + *SE*_*A*_ + *SE*_*MR*_, in which the third term is very small and can be neglected.

The S-parameter measured using the vector network analyzer is related to the reflection and transmission coefficient as follows: the transmission (T) equals the squared of the absolute values of S_12_ or S_21_ and the refection (R) equals the squired of the absolute value of S_11_ or S_22_. The total shielding effectiveness can be calculated as the sum of the refection and absorption values and related to the S-parameters as^50^:

$${SE}_{TOT}= -10 {log}_{10}{\left|{S}_{12}\right|}^{2}$$

The electromagnetic shielding effectiveness of all prepared samples was measured in the x-band range from 8 to 12 GHz. The response of all samples behaves the same pattern as each one has different local maxima and minima. There are three local maxima around 9.25 GHz, 10.25 GHz and 11.25 GHz with the third one has the largest value.

**Figure 14:**
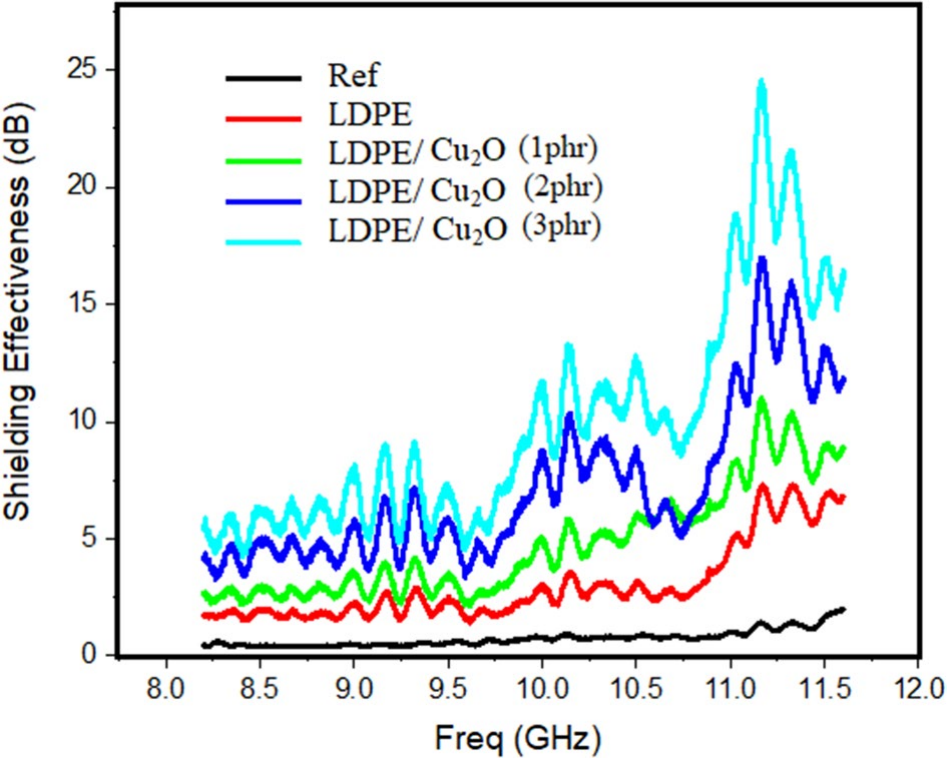
Represents the Shielding Effectiveness in (dB) versus frequency in (GHz) for the unirradiated samples.

**Figure 15:**
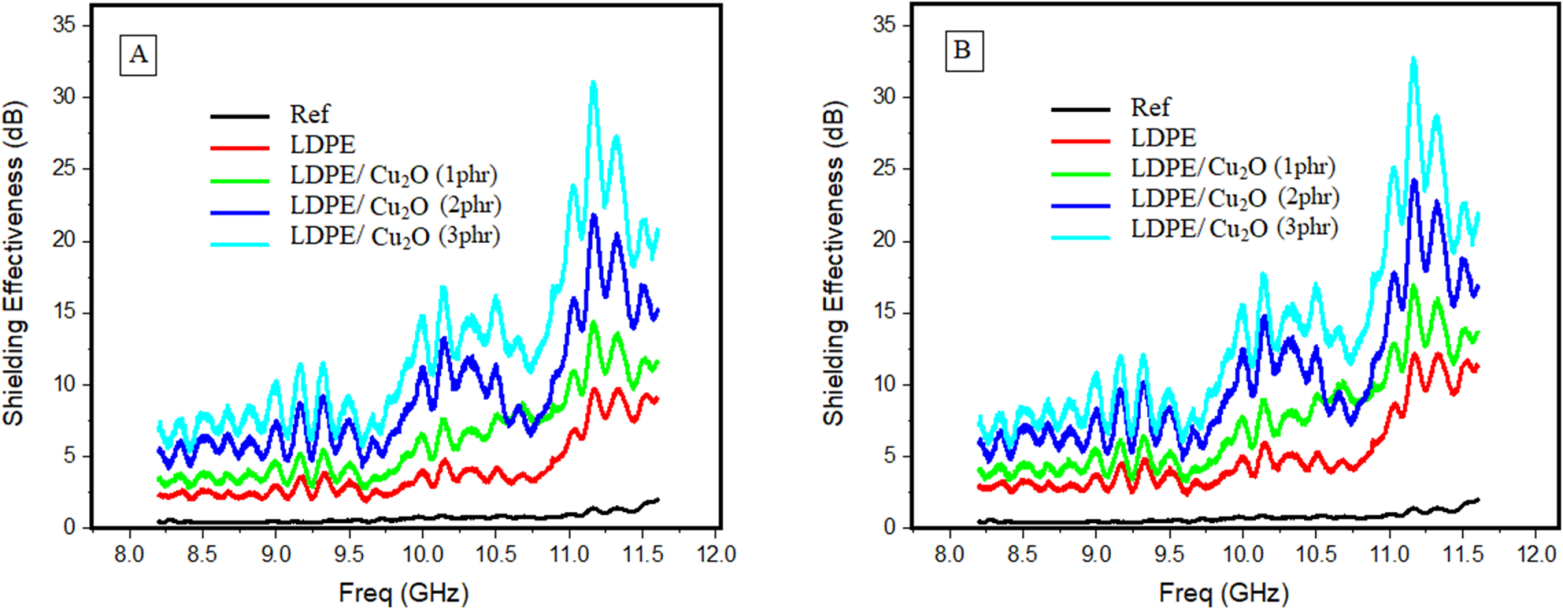
Represents the Shielding Effectiveness in (dB) vs. frequency in (GHz) for (**A**) 50 kGy and (**B**) 100 kGy of gamma-ray irradiated samples.

**Figure 16:**
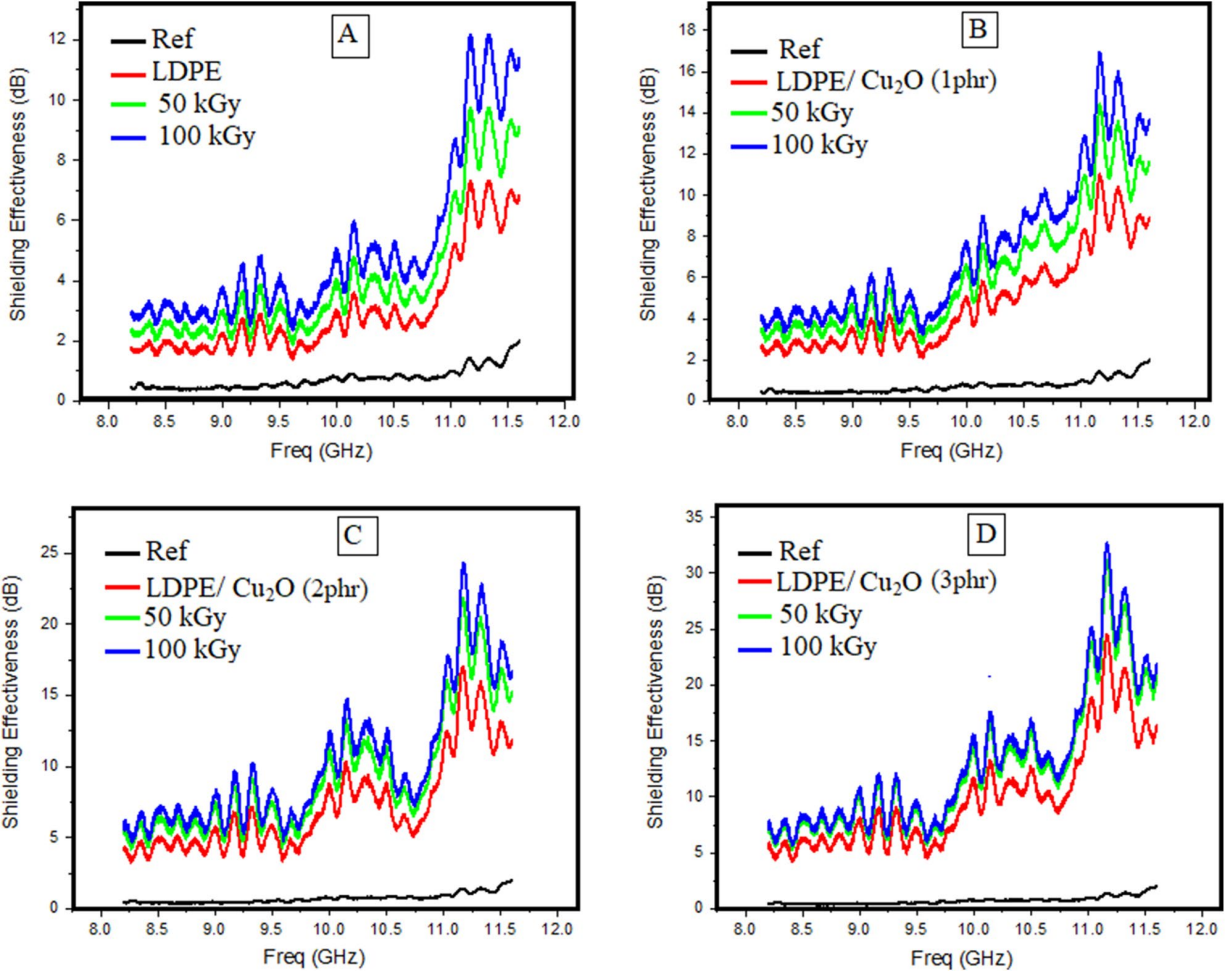
Represents the SE of each prepared sample before and after gamma-ray irradiation with 50 and 100 kGy respectively, (**A**) LDPE, (**B**) LDPE/Cu_2_O (1phr) nanocomposite, (**C**) LDPE/Cu_2_O (2 phr) nanocomposite and (**D**) LDPE/Cu_2_O (2 phr) nanocomposite .

The shielding effectiveness of the LDPE/Cu_2_O nanocomposite samples before irradiations is presented in Fig. 14. By investigating Figs. 14, 15 and 16, the Ref curve (represented by the black line) regards to measurement of the shielding effectiveness without any obstacles to be considered as a reference measurement (datum curve) for all sample. The LDPE curve (represented by the red line) represents the measurement of the control sample without Cu_2_O nanoparticles. The remained curves represent the samples with the addition of the effective material with different concentrations under study. The shielding effectiveness increased as the percentage of the LDPE/Cu_2_O nanocomposite increased in the sample.

The shielding effectiveness of the LDPE/Cu_2_O nanocomposite samples after 50 kGy of gamma-ray irradiation is presented in Fig. 15A. The response is similar to that of the unirradiated samples but the shielding effectiveness improved significantly. On the other hand, with increasing gamma radiation to 100 kGy (Fig. 15B), the response is similar to both the unirradiated and irradiated with 50 kGy samples but the shielding effectiveness improved significantly. The enhancing EMI shielding process is attributed to the enhancement of the conductivity by gamma radiation and Cu_2_O nanoparticles on LDPE polymeric matrix^51–53^**.**

A study of the radiation effect on each sample is presented in Fig. 16. Each graph in this figure represents the response of each sample before and after irradiation. It is clear that the increase in the radiation dose enhances the shielding effectiveness of the prepared samples. The shielding effectiveness improved significantly from 25 dB for unirradiated samples to 35 dB when irradiated with 100 kGy, which reflects 40% enhancement in the effectiveness of the shielding.

## Conclusions

This article presented the synthesis and investigation of gamma irradiated LDPE/Cu_2_O nanocomposites. TEM and XRD investigations proved that the Cu_2_O nanoparticles were successfully formed with particle size equal 16.8 nm. Based on the mechanical results, we conclude that the Cu_2_O nanoparticles positively tensile test results on LDPE matrix at 2 phr Cu_2_O nanoparticles and 100 kGy. SEM results show a homogeneous dispersion of nanofillers inside LDPE matrix. From TGA analysis, the thermal stability of LDPE/Cu_2_O nanocomposites clearly improved with all Cu_2_O percentages. The shielding effectiveness was measured for unirradiated and irradiated nanocomposites with gamma radiation doses (50 and 100 kGy). The results of SE increase significantly with the increase of both the concentration of Cu_2_O nanoparticles and the radiation doses. In conclusion, the findings of our investigation witness the remarkable scope and potency of LDPE/Cu_2_O nanocomposites as efficient product for electromagnetic interference (EMI) shielding and radiation pollution which lead to the detrimental effects on sensitive precision electronics and on human health.

## Data Availability

All data generated or analyzed during this study are available from the corresponding author on request.
